# Determinants of hospitalization expenses in primary arthroscopic stabilization for recurrent shoulder anterior instability: A retrospective analysis

**DOI:** 10.1097/MD.0000000000049165

**Published:** 2026-06-05

**Authors:** Jian Xue, Jian Wang, Jinchun Wu, Jie Zhao

**Affiliations:** a Department of Orthopedics, Guangdong Provincial Corps Hospital of Chinese People’s Armed Police Force, Guangzhou, P. R .China; b Cancer Prevention Center, State Key Laboratory of Oncology in South China, Guangdong Provincial Clinical Research Center for Cancer, Sun Yat-sen University Cancer Center, Guangzhou, P. R. China.

**Keywords:** cost variation, hospitalization expenses, primary arthroscopic stabilization, recurrent shoulder anterior instability

## Abstract

To identify the factors affecting hospital cost associated with primary arthroscopic stabilization for recurrent shoulder anterior instability. The clinical data of patients with symptoms of recurrent glenohumeral anterior stability who underwent arthroscopic stabilization at a single institution between January 2020 and April 2022 were collected retrospectively. Surgical information and patient characteristics were recorded. Variations in hospital hospitalization expenses were identified and extracted from the medical and billing record keeping system at the hospital. Sixty-one cases of recurrent anterior instability of the glenohumeral joint were identified in 59 patients during the study period. There were no significant differences in hospitalization expenses between the Arthroscopic Bankart repair and Arthroscopic Latarjet groups (*P* = .624), nor across the different periods (*P* = .54). However, compared with the minority group, the majority group required a higher number of surgical procedures (1 [1, 1.5] vs 2 [1, 2], *P* = .011) and had longer operative durations (120.00 [107.00, 135.00] minutes vs 162.50 [130.00, 222.50] minutes, *P* = .001). Total hospitalization expenses were significantly higher in the majority group (10,144.23 ± 1795.78 United States dollar [USD]) than in the minority group (9042.87 ± 943.22 USD) (*P* = .003), driven by increased costs for implants (3848.84 ± 855.72 USD vs 4623.81 ± 1175.04 USD, *P* = .005), consumables (6886.56 ± 1013.82 USD vs 7649.36 ± 1620.16 USD, *P* = .028), anesthesia (371.98 [357.98, 379.40] USD vs 400.82 [371.98, 429.66] USD, *P* = .001), medications (524.45 [427.86, 631.29] USD vs 623.39 [515.35, 824.74] USD, *P* = .02), and anesthetics (168.83 ± 44.55 USD vs 213.83 ± 66.82 USD, *P* = .003). No significant differences were observed between the minority and majority groups in operation expenses, non-implanted consumables, perioperative drug costs, nursing and examination fees, or days of hospitalization (all *P* > .05). Patients who experienced 3 or more recurrent dislocations prior to primary arthroscopic stabilization incurred significantly higher hospitalization expenses, driven by increased implant use and prolonged operative time. These findings suggest that earlier surgical intervention in patients with recurrent anterior shoulder instability may help reduce procedural complexity and associated costs.

## 1. Introduction

Owing to its wide range of motion and relatively low stability, the glenohumeral joint is prone to anterior dislocation.^[1]^ Statistics show that the overall incidence rate of glenohumeral dislocation varies between 15.3 and 56.3 per 100,000 people annually.^[2–4]^ According to current estimates, the likelihood of glenohumeral dislocation is higher in military personnel than in civilians.^[5,6]^ This susceptibility to instability may increase the risk of recurrence, affecting as many as 46.6% to 90% of those who have experienced initial anterior glenohumeral dislocation and received nonoperative treatment.^[7–10]^

Compared with nonoperative treatment modalities, surgery is considered a more appropriate intervention for correcting anterior glenohumeral instability because of its association with a lower risk of recurrence.^[8,11,12]^ Moreover, according to current literature, surgical stabilization can reduce overall costs as well as decrease the risk of palindromic redislocation.^[13,14]^

Although the optimal surgical treatment is still controversial, arthroscopic stabilization of the shoulder joint causes minimal soft tissue damage,^[15]^ is performed in approximately 90% of cases,^[16]^ and has been the long-standing first-line treatment choice.^[17,18]^ Two commonly employed arthroscopic techniques are the Bankart repair and the Latarjet procedure. Although several studies^[19,20]^ have identified the open Latarjet procedure as a dominant and cost-effective approach for first-time shoulder dislocations, these investigations did not directly compare hospitalization or surgical-related costs. However, the average cost of arthroscopic stabilization ranged from $15,287^[21]^ to $20,385,^[22]^ making it a focal point for healthcare policy and patients alike. Moreover, few studies have focused on the factors affecting the cost arthroscopic stabilization. In a retrospective analysis, Peter N. Chalmers et al examined patients who underwent arthroscopic surgery for glenohumeral instability and noted that most perioperative expenditures can be directly attributed to the costs of implants and facility utilization.^[23]^ Although existing cost analyses for anterior shoulder instability have largely focused on comparing surgical with nonsurgical management,^[24,25]^ the relationship between instability severity and the cost of primary arthroscopic stabilization remains unclear. Specifically, a higher number of recurrent dislocations may be associated with progressive intra-articular pathology, which can necessitate additional surgical steps and increased implant use, potentially contributing to higher hospitalization costs. Furthermore, temporal factors (such as the surgeon’s learning curve or different time periods) may also influence costs, although such time-related analyses remain scarce in this context. That is, a related issue that is not fully understood is the influence of instability severity on the factors affecting the cost of primary arthroscopic stabilization.

The purpose of this study was to identify the factors affecting the cost of hospitalization associated with primary arthroscopic stabilization for recurrent shoulder anterior instability. We hypothesized that the cost of hospitalization for patients who undergo primary arthroscopic stabilization might be greater for those who frequently experience recurrent shoulder anterior instability.

## 2. Methods

### 2.1. Study design

This retrospective cross-sectional study was performed at a single institution. Every patient provided written consent. All methods were performed in accordance with the relevant guidelines and regulations. The Ethics Committee of Guangdong Provincial Corps Hospital of Chinese People’s Armed Police Force has confirmed that no ethical approval is required.

### 2.2. Patient selection

In this study, we evaluated military patients who underwent primary arthroscopic stabilization between January 2020 and April 2022. Patients who were selected for inclusion had to meet all the following inclusion criteria: visited our hospital due to persistent symptoms associated with recurrent glenohumeral anterior instability; had undergone unilateral shoulder surgery; underwent perioperative evaluations and postoperative management by the same professional medical team; and underwent the arthroscopic procedure by the same senior lead surgeon. We excluded patients who met any of the following exclusion criteria: underwent revision surgery or had a history of any operation for former dislocations, had a first-time shoulder dislocation, developed an infection during hospitalization, or had rotator cuff tears or subacromial impingement syndrome that needed additional surgical intervention.

### 2.3. Arthroscopic surgery procedure

Patients were secured in the lateral recumbent position after general anesthesia induction. Arthroscopic Bankart repair, a solely soft-tissue procedure, was performed for those with defects involving 25% or less of the anterior–inferior glenoid osseous. Arthroscopic Latarjet procedure with 2 endobuttons was selected for the bony Bankart lesions involving >25% of the anterior–inferior glenoid osseous. Arthroscopic procedures were also required for concomitant type Ⅱ, Ⅲ or Ⅳ Superior Labrum Anterior and Posterior lesions. A Remplissage procedure was performed for off-track Hill-Sachs lesions, if present.

The TWINFIX Ti Preloaded Suture Anchor was used for all operations. The grafted bone was fixed to the glenoid using suture anchors and an ENDOBUTTON^◇^ CL ULTRA Fixation Button with 20 mm continuous loop suture.

The duration of surgery was recorded to estimate the difficulty of surgery.

### 2.4. Cost data collection

We extracted cost data from the medical and billing record keeping system at the hospital. Data were collected in accordance with relevant published costing guidelines,^[26]^ and any necessary changes were made. Searches and data extractions were analyzed for the time horizons of January 2020 and April 2022. Follow-up duration or timing of postoperative assessment was not included as part of the study design or the inclusion criteria, as the present analysis focused exclusively on hospitalization costs incurred during the index admission.

In addition to total hospitalization expenses, medical items that patients were directly responsible for were collected for analysis. These items mainly included medication and consumables. More precisely, anesthetics, perioperative drugs, implants, and nonimplanted consumables were identified and valuated Operating room costs, including the costs of anesthesia and the operation itself, were also calculated in our study. Moreover, we included potential confounders that might affect hospitalization expenses, such as days of hospitalization, nursing care and examination fees. Overhead costs including the costs of patient travel, follow-up, rehabilitation, and lost wages, which are unquantifiable and immeasurable, were not considered.

All costs were converted from Chinese Yuan and presented as United States dollar (USD) from an exchange rate of Chinese Yuan 1 = USD 0.14.

### 2.5. Study group

Different surgical methods and time trends could be confounding factors affecting price and surgical technique, which may influence hospitalization expenses and the duration of surgery. First, the surgical shoulders were separated into 2 groups: the Bankart repair group (the arthroscopic Bankart repair technique) and the Latarjet group (the arthroscopic Latarjet procedure). The surgical shoulders were then divided into 3 groups on average according to the operation date.

Finally, all the chosen shoulders in this research were classified into 2 groups based on the number of glenohumeral dislocations: the shoulders with fewer than or equal to 3 recurrent glenohumeral anterior dislocations were included in the minority group; otherwise, the shoulders were categorized into the majority group.

### 2.6. Statistical analysis

All the data were collected by 2 authors independently and entered into Excel (Microsoft Excel 2019MSO) to assist with data management and tabulation. IBM SPSS Statistics v26.0 (IBM Corp., New York) was used for all statistical analyses. Normally distributed quantitative data are presented as means ± standard deviation and were compared using the independent Student *t* tests. Continuous variables with a nonnormal distribution are expressed as medians (interquartile range) and were analyzed by the Mann–Whitney *U* test. The Kruskal–Wallis test was used to compare multiple groups. When the overall test yielded a statistically significant result (*P* < .05), all pairwise comparisons between groups were performed, and the Bonferroni correction was applied to adjust for multiple comparisons. Categorical data are presented as counts and percentages of the total. Statistical significance is taken at *P < .05 and **P < .01.

## 3. Results

### 3.1. Description of demographics

**Figure 1.**
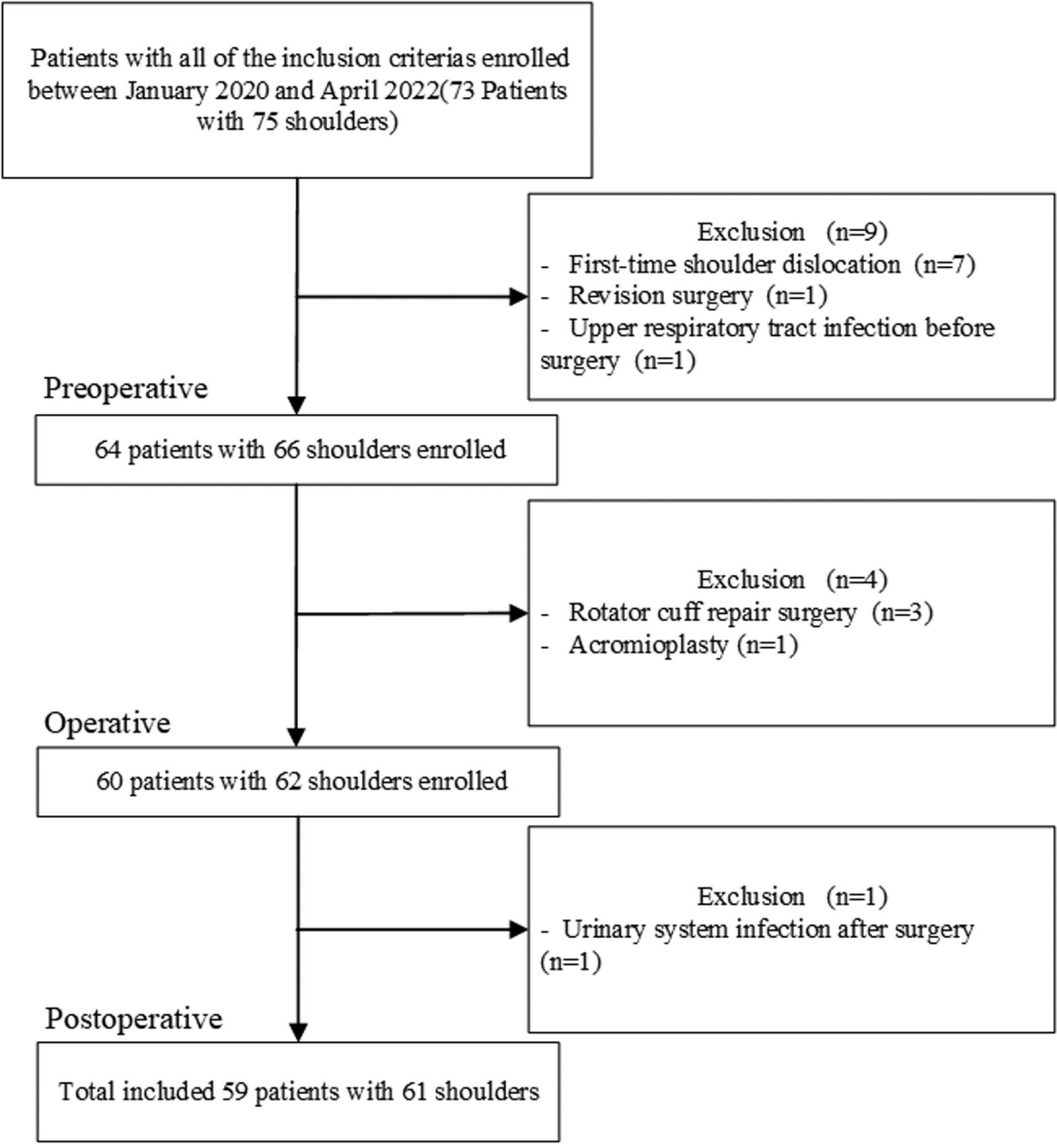
Flowchart of patient recruitment shows the flowchart of patient inclusion and exclusion. A total of 73 patients with 75 shoulders met the inclusion criteria between January 2020 and April 2022. 14 patients were excluded due to first-time dislocation, perioperative infection, revision surgery, or concomitant rotator cuff repair or acromioplasty, leaving 59 patients with 61 shoulders for analysis.

Figure 1 shows the flowchart of patient inclusion and exclusion. During the period from January 2020 to April 2022, 73 potentially eligible patients with 75 shoulders were identified. Among these patients, 14 (19%) with a first-time glenohumeral dislocation (n = 7), who developed an infection during hospitalization (n = 2), who underwent revision surgery (n = 1), and required additional steps for rotator cuff repair or acromioplasty (n = 4) were excluded. A total of 61 shoulders of 59 patients were ultimately included.

The average age of the patients was 23.51 ± 4.14 years. Equal numbers of right (45.76%) and left shoulders (50.85%) were selected for surgery. Two patients (3.39%) underwent staging surgery for both shoulders. Eleven shoulders (18.03%) underwent arthroscopic Latarjet, whereas 50 shoulders (81.97%) underwent arthroscopic Bankart repair. With respect to the number of procedures, more than half of the shoulders (54.10%) underwent 1 procedure; 26 shoulders (42.62%) underwent 2 procedures, whereas the rest underwent 3 procedures (2 shoulders, 3.28%). The clinical characteristics and demographics of the final population are presented in Table 1.

**Table 1:** Clinical characteristics and demographics.

Variable	n	%	Mean ± SD	Range
Age at surgery (yr)			23.51 ± 4.14	19–37
Gender				
Male	59	72.88		
Female	16	27.12		
Marital status				
Unmarried	52	88.14		
Married	7	11.86		
Operated-side shoulder				
Left only	30	50.85		
Right only	27	45.76		
Both	2	3.39		
Duration of shoulder problems				
<6 month	8	13.11		
6 month to 1 year	23	37.71		
1–2 years	20	32.79		
>2 years	10	16.39		
Major procedure				
Bankart repair	50	81.97		
Iliac crest bone grafting	11	18.03		
Additional procedure				
SLAP repair	21	34.43		
Remplissage	8	13.11		
Number of procedure				
1	33	54.1		
2	26	42.62		
3	2	3.28		

SD = standard deviation, SLAP = Superior Labrum Anterior and Posterior.

### 3.2. Comparison between the Arthroscopic Bankart repair and Arthroscopic Latarjet groups

Table 2 shows the differences in total costs between the arthroscopic Bankart repair group and the arthroscopic Latarjet group. We observed that operative duration (Bankart 130.00 [119.00, 171.25] minutes vs Latarjet [150.00, 245.00] minutes, *P* = .002), operation expenses (Bankart 643.51 [643.51, 643.51] USD vs Latarjet 828.06 [643.51, 894.31] USD, *P* = .001), anesthesia fee (Bankart 371.98 [371.98, 401.28] USD vs Latarjet 400.82 [375.62, 433.86] USD, *P* = .041), anesthetic medication cost (Bankart 190.88 ± 8.33 USD vs Latarjet 232.22 ± 22.65 USD, *P* = .049), and nursing and examination fees (Bankart 625.86 [499.10, 748.90] USD vs Latarjet 805.88 [766.34, 851.57] USD, *P* = .001) were greater in the arthroscopic Latarjet group than in the arthroscopic Bankart repair group. However, the small differences in the other comparison items were not statistically significant.

**Table 2:** Cost variability for different surgical methods.

Cost variability†	Bankart repair (n = 50, 81.97%)	Latarjet (n = 11, 18.03%)	*T*/*U*-value	*P*-value
Age at surgery (yr)	22 (21, 24)	22 (20, 25)	272.50	.962
Days of hospitalization (d)	14.66 ± 0.54	15.36 ± 0.91	‐0.22	.568
Operative duration (min)	130.00 (119.00, 171.25)	190.00 (150.00, 245.00)	111.50	.002**
Surgical procedures (n)	1 (1, 2)	1 (1, 2)	239.00	.440
Hospitalization expenses (USD)	9691.11 ± 191.63	10,101.28 ± 790.76	‐0.50	.624
Anesthesia (USD)	371.98 (371.98, 401.28)	400.82 (375.62, 433.86)	168.50	.041*
Operation expenses (USD)	643.51 (643.51, 643.51)	828.06 (643.51, 894.31)	133.50	.001**
Consumables (USD)	7386.23 ± 178.03	7389.17 ± 696.00	-0.04	.997
Implants (USD)	4308.61 ± 157.71	4577.08 ± 371.43	‐0.71	.481
Non-implanted consumables (USD)	2957.71 (2790.35, 3226.72)	3141.89 (2156.03, 3309.38)	266.00	.866
Medication (USD)	555.70 (452.48, 768.28)	614.69 (557.91, 957.67)	193.00	.124
Anesthetics (USD)	190.88 ± 8.33	232.22 ± 22.65	‐2.00	.049*
Perioperative drug (USD)	375.71 (279.77, 536.51)	352.02 (284.78, 756.42)	257.00	.736
Nursing and examination fees (USD)	625.86 (499.10, 748.90)	805.88 (766.34, 851.57)	102.00	.001**

† Normally-distributed quantitative data are displayed as mean ± standard deviation and were compared by the independent Student *t* test (*T*). Continuous variables with non-normal distribution are displayed as median (interquartile range) and analyzed by the Mann–Whitney *U* test (*U*).

* *P* < .05.

** *P* < .01.

### 3.3. Comparison in different periods

To test whether the learning curve or different periods affected hospitalization expenses, the selected shoulders were subdivided into tertiles (T1, T2 and T3) depending on the operation date: T1, from January 1, 2020 to 2 July 2, 2020, n = 20; T2, from July 3, 2020 to June 15, 2021, n = 20; and T3, from June 16, 2021 to April 30, 2022, n = 21 (Table 3). Comparisons between the different tertiles and the results in Table 3 confirmed that the means of the average costs of anesthetics and nonimplanted consumables at T3 were significantly different from those at T1 and T2, respectively. However, the costs of medication and consumables across the 3 groups did not differ significantly. Although nursing and examination fees were significantly lower in T1 (560.29 [457.49, 651.31] USD) than in T2 (727.03 [532.65, 855.53] USD) and T3 (744.59 [594.34, 822.25] USD) (*P* = .003), no significant difference was observed in total hospitalization expenses across the 3 periods (T1: 9834.84 ± 1607.93 USD; T2: 9940.86 ± 1676.10 USD; T3: 9531.21 ± 1671.84 USD; *P* = .54). Moreover, differences in age, surgical duration, and other cost factors between the different groups were not statistically significant.

**Table 3:** Comparison of cost variability in different periods.

Cost variability†	T1 (n = 20, 32.79%)	T2 (n = 20, 32.79%)	T3 (n = 21, 34.42%)	*χ* ^2^	*P*-value
Age at surgery (yr)	22.5 (20.25, 23.75)	22 (21.00, 24.75)	22 (21.00, 26.00)	0.39	.82
Days of hospitalization (d)	13.45 ± 2.84	15.40 ± 3.68	15.48 ± 4.12	3.52	.17
Operative duration (min)	150.00 (120.00, 177.50)	140.00 (120.00, 215.50)	140.00 (110.00, 185.00)	0.31	.86
Surgical procedures (n)	1.5 (1.0, 2.0)	1.5 (1.0, 2.0)	1 (1.0, 2.0)	0.60	.74
Hospitalization expenses (USD)	9834.84 ± 1607.93	9940.86 ± 1676.10	9531.21 ± 1671.84	1.23	.54
Anesthesia (USD)	391.72 (371.98, 423.50)	376.18 (371.98, 405.02)	371.98 (350.56, 401.73)	2.80	.25
Operation expenses (USD)	643.51 (643.51, 643.51)	643.51 (643.51, 757.08)	643.51 (643.51, 778.37)	5.93	.051
Consumables (USD)	7608.09 ± 1547.61	7530.25 ± 1504.92	7039.31 ± 1387.02	2.76	.25
Implants (USD)	4620.21 ± 1098.01	4263.32 ± 1049.32	4195.60 ± 1241.53	1.30	.52
Non-implanted consumables (USD)	2968.44 (2890.93, 3260.32)	3174.45 (2908.98, 3758.95)	2887.27 (2730.75, 3059.53)§	7.29	.03*
Medication (USD)	642.73 (462.75, 822.45)	537.57 (432.81, 665.88)	561.75 (488.04, 763.86)	1.31	.52
Anesthetics (USD)	147.70 (127.03, 205.92)	192.81 (162.35, 225.96)	220.56 (173.40, 282.02)‡	10.44	.005**
Perioperative drug (USD)	455.52 (330.79, 703.69)	317.78 (231.15, 478.48)	316.66 (282.10, 546.05)	3.71	.16
Nursing and examination fees (USD)	560.29 (457.49, 651.31)	727.03 (532.65, 855.53)‡	744.59 (594.34, 822.25)‡	11.64	.003**

† Multiple comparisons were performed by Kruskal–Wallis analysis (*χ*^2^). For variables with a statistically significant Kruskal–Wallis test (*P* < .05), all pairwise comparisons were performed with Bonferroni correction.

‡ Statistically significant difference compared with T1 after Bonferroni correction.

§ Statistically significant difference compared with T2 after Bonferroni correction.

* *P* < .05.

** *P* < .01.

### 3.4. Comparisons based on the number of recurrent anterior instability episodes

As shown in Table 4, the 61 operative shoulders were further classified into 2 groups according to the number of anterior instability recurrences: the minority group (≤3 recurrent glenohumeral anterior dislocations) and the majority group (>3 recurrent glenohumeral anterior dislocations). Compared with the minority group, the majority group had a higher number of surgical procedures (minority 1 [1, 1.5] vs majority 2 [1, 2], *P* = .011) and greater implant costs (minority 3848.84 ± 855.72 USD vs majority 4623.81 ± 1175.04 USD, *P* = .005), suggesting more frequent structural damage requiring repair, which in turn affected consumable costs (minority 6886.56 ± 1013.82 USD vs majority 7649.36 ± 1620.16 USD, *P* = .03). Moreover, the operative duration was 162.50 (130.00, 222.50) minutes in the majority group and 120.00 (107.00, 135.00) minutes in the minority group (*P* = .001). Consequently, anesthesia fees (minority 371.98 [357.98, 379.40] USD vs majority 400.82 [371.98, 429.66] USD, *P* = .001), medication costs (minority 524.45 [427.86, 631.29] USD vs majority 623.39 [515.35, 824.74] USD, *P* = .02), and anesthetic costs (minority 168.83 ± 44.55 USD vs majority 213.83 ± 66.82 USD, *P* = .003) were significantly higher in the majority group. Notably, total hospitalization expenses were also significantly higher in the majority group (minority 9042.87 ± 943.22 USD vs majority 10,144.23 ± 1795.78 USD, *P* = .003), whereas no significant differences were observed in days of hospitalization (minority 14.33 ± 3.68 days vs majority 15.03 ± 3.67 days, *P* = .49) or other cost components between the 2 groups.

**Table 4:** Cost variability for minority and majority groups.

Nursing and examination fees	Minority (n = 21, 34.43%)	Majority (n = 40, 65.57%)	*T*/*U*-value	*P*-value
Age at surgery (yr)	21 (20, 23)	22.5 (22, 25)	535.50	.08
Days of hospitalization (d)	14.33 ± 3.68	15.03 ± 3.67	‐0.70	.49
Operative duration (min)	120.00 (107.00, 135.00)	162.50 (130.00, 222.50)	645.00	.001**
Surgical procedures (n)	1 (1, 1.5)	2 (1, 2)	566.50	.011*
Hospitalization expenses (USD)	9042.87 ± 943.22	10,144.23 ± 1795.78	‐3.14	.003**
Anesthesia (USD)	371.98 (357.98, 379.40)	400.82 (371.98, 429.66)	629.00	.001**
Operation expenses (USD)	643.51 (643.51, 643.51)	643.51 (643.51, 707.39)	483.00	.25
Consumables (USD)	6886.56 ± 1013.82	7649.36 ± 1620.16	‐2.25	.03*
Implants (USD)	3848.84 ± 855.72	4623.81 ± 1175.04	‐2.94	.005**
Non-implanted consumables (USD)	2891.72 (2694.93, 3201.24)	2983.22 (2890.97, 3286.87)	493.00	.27
Medication (USD)	524.45 (427.86, 631.29)	623.39 (515.35, 824.74)	576.00	.02*
Anesthetics (USD)	168.83 ± 44.55	213.83 ± 66.82	‐3.13	.003**
Perioperative drug (USD)	318.27 (249.66, 480.69)	378.24 (290.82, 706.45)	502.00	.21
Nursing and examination fees (USD)	621.71 (431.30, 728.61)	688.82 (570.62, 821.05)	539.00	.07

† Normally-distributed quantitative data are displayed as mean ± standard deviation and were compared by the independent Student *t* test (*T*). Continuous variables with non-normal distribution are displayed as median (interquartile range) and analyzed by the Mann–Whitney *U* test (*U*).

* *P* < .05.

** *P* < .01.

## 4. Discussion

In this study, by analyzing the costs of hospitalization associated with primary arthroscopic surgery in 61 shoulders at a single medical institution, our observations revealed that patients who experienced more shoulder anterior dislocations (>3 dislocations) incurred significantly longer operative times and higher hospital costs than did those with fewer symptoms. Specifically, the majority group had a longer operative duration (162.50 [130.00, 222.50] minutes vs 120.00 [107.00, 135.00] minutes, *P* = .001) and higher total hospitalization expenses (10,144.23 ± 1795.78 USD vs 9042.87 ± 943.22 USD, *P* = .003) compared with the minority group. These findings may suggest that the longer operative time and greater number of primary arthroscopic stabilization procedures, as well as the greater hospitalization cost, were likely due to the lack of early surgical intervention for recurrent glenohumeral anterior instability until the number of shoulder dislocations increased (>3 times). These most important findings in this paper validate our previously proposed hypothesis. However, we evaluated different surgical methods or periods and found no statistically significant difference in total hospitalization expenses (Bankart repair: 9691.11 ± 191.63 USD vs Latarjet: 10,101.28 ± 790.76 USD, *P* = .624; T1: 9834.84 ± 1607.93 USD, T2: 9940.86 ± 1676.10 USD, T3: 9531.21 ± 1671.84 USD, *P* = .54), meaning that neither different surgical methods nor the learning curve were significant factors affecting cost. Moreover, this finding showed that the period when the price was maintained at a relatively steady level may not have been the main driver influencing hospitalization expenses.

Anterior glenohumeral dislocations in particular populations, such as young athletes and military individuals, may be a common complaint upon presenting to the emergency departments.^[6,27]^ It is usually successful for an emergency doctor to reduce the dislocated shoulder immediately after a thorough examination.^[28]^ Although numerous studies have supported early surgical intervention for primary shoulder dislocation,^[29–31]^ some patients still choose nonsurgical treatment because of immediate pain relief and recovery of range of motion after close reduction. However, the most common complication of anterior shoulder dislocation after nonoperative treatment is recurrence, especially in the young population.^[8]^ Given that not all patients with recurrent dislocation are comfortable with timely surgical treatment, according to the literature,^[32]^ the median number of dislocations was 3, which was used as the cutoff value for our study grouping.

It is not uncommon to encounter a variety of shoulder lesions that can be found in patients with recurrent shoulder anterior instability.^[33]^ Yiannakopoulos et al^[7]^ arthroscopically ascertained 127 patients with acute or chronic anterior glenohumeral instability and reported that the incidence of Bankart lesions was 97.11% and that of Hill-Sachs lesions was 93.26% in chronic patients. They concluded that the number of pathological shoulder lesions tends to increase, probably due to the increase in the number of dislocations in chronic patients with recurrent shoulder anterior dislocations. Hantes et al^[34]^ reached the same conclusion and noted that the incidence of shoulder lesions, such as Bankart, bony Bankart, Superior Labrum Anterior and Posterior and Hill-Sachs lesions, increases with time in shoulders with chronic recurrent anterior instability. Our findings seem to be in line with these results, thus adding to the previous literature. In this study, we observed that the majority group was subjected to more surgical steps (2 [1–2] vs 1 [1–1.5], *P* = .011), had longer procedure times (162.50 [130.00, 222.50] minutes vs 120.00 [107.00, 135.00] minutes, *P* = .001) and spent more on implants (4623.81 ± 1175.04 USD vs 3848.84 ± 855.72 USD, *P* = .005), indicating that the lesions in the shoulder probably increased in severity as the number of recurrent dislocations increased. Each additional surgical step prolongs the surgery duration and requires additional implants.^[23]^ This likely, in part, explains the higher costs in the majority group.

Our study clearly confirmed that consumable cost was a major factor driving hospitalization expenses, with significantly higher consumable costs observed in the majority group (7649.36 ± 1620.16 USD vs 6886.56 ± 1013.82 USD, *P* = .028). This finding could correspond with the findings of earlier studies.^[23,35]^ Uffmann et al^[35]^ compared cost variance between the arthroscopic Bankart procedure and the Latarjet procedure using univariate analysis. They demonstrated that cost variance in arthroscopic Bankart repair was solely driven by implant-related costs, along with supplies and suture anchors. In additionto consumable cost, our findings further suggest that procedure time may be another important cost driver that should not be neglected. Indeed, similar observations were also made in several recent studies. In determining the cost-effectiveness of arthroscopic rotator cuff repair, Burns et al noted that reducing the amount of time in the operating room time may provide appreciable benefits for cost analyses. Patients may experience cost reductions related to fewer anesthetic side effects and less pain after surgery.^[36]^ Li et al also reported that costs increased, mainly because of the longer operative times, when they analyzed 3 different surgical methods for shoulder dislocation from 6 states in the 2014 State Ambulatory Surgery and Services Databases.^[37]^

Although the optimal surgical techniques for recurrent anterior shoulder instability remain unknown, arthroscopic stabilization is preferred because of its outcomes.^[38]^ Furthermore, surgical shoulder stabilization is a substantial medical expense for both medical insurance companies and patients, and there are few studies on the relationship between the severity of recurrent shoulder instability and hospital costs associated with primary arthroscopic stabilization surgery.

According to previous studies, arthroscopic surgery for recurrent shoulder anterior instability has advantages over nonoperative management in terms of a lower rate of recrudescence and better cost-effectiveness. Hurley et al^[39]^ conducted a meta-analysis of 10 prospective studies and reported that the recurrence rate associated with arthroscopic Bankart repair was 7-fold lower than that associated with nonoperative approaches. Using the Markov model to analyze 6 models over 15 years, Crall et al compared surgical outcomes with nonoperative outcomes in 1000 patients whose shoulder was anteriorly dislocated for the first time. They suggested that arthroscopic stabilization is more effective and more cost-effective.^[40]^ Similarly, on the basis of an analysis of data from 104 with dislocated shoulders, Comadoll et al concluded that stabilization surgery is helpful for reducing health care costs.^[41]^ The findings of our study align with those of studies on hospitalization costs associated with primary arthroscopic surgery for recurrent shoulder anterior instability. Statistically significant differences in cost variation were observed between the minority group and the majority group across multiple cost categories, including implant costs, consumable expenses, anesthesia fees (400.82 [371.98, 429.66] USD vs 371.98 [357.98, 379.40] USD, *P* = .001), medication costs (623.39 [515.35, 824.74] USD vs 524.45 [427.86, 631.29] USD, *P* = .02), anesthetic costs (213.83 ± 66.82 USD vs 168.83 ± 44.55 USD, *P* = .003), and total hospitalization expenses (*P* = .003), as detailed in Table 4. These results suggest that early arthroscopic stabilization is beneficial for reducing patient healthcare expenditures and alleviating social and economic financial burdens.

## 5. Limitations

There are several limitations that need to be addressed. The greatest limitation is the small sample size. The aim of this study was to explore the impact of disease severity on hospitalization costs. Since the best operative management method for recurrent shoulder anterior instability is still unknown, we selected arthroscopic stabilization for this study because of its extensive clinical applications. To avoid variations in prices and surgical team preferences across different areas, we set strict inclusion and exclusion criteria. To reduce the possibility of postoperative variability, we ensured that there was minimal variation in the protocol. Thus, these factors resulted in a smaller sample size. We acknowledge that the small sample size may increase the potential for type I errors. An additional limitation of this study is its retrospective cross-sectional study design. We were unable to follow patients who visited another hospital to further assess the costs association with rehabilitation therapy. Consequently, this could undervalue the overall treatment burden of primary arthroscopic surgery for recurrent shoulder anterior instability. A third limitation is the absence of recovery times. The costs of recovery in the groups could not be evaluated or compared. In addition, indirect costs, such as transportation expenses, nursing expenses, and time off from work, were not considered in the study. Typically, these costs, which can be substantial, are difficult to estimate with any degree of accuracy. However, the findings of the current study are promising and might serve as a reference and basis for future health care insurance decision-making.

## 6. Conclusions

Patients who experienced 3 or more recurrent dislocations prior to primary arthroscopic stabilization incurred significantly higher hospitalization expenses, driven by increased implant use and prolonged operative time. These findings suggest that earlier surgical intervention in patients with recurrent anterior shoulder instability may help reduce procedural complexity and associated costs.

## Author contributions

**Data curation:** Jian Xue, Jian Wang, Jinchun Wu.

**Formal analysis:** Jian Wang, Jinchun Wu.

**Investigation:** Jian Xue, Jian Wang, Jinchun Wu.

**Methodology:** Jian Xue, Jie Zhao.

**Project administration:** Jian Xue, Jian Wang.

**Supervision:** Jian Xue, Jie Zhao.

**Writing – original draft:** Jie Zhao.

**Writing – review & editing:** Jie Zhao.
